# Supplementary material for: Pericyte-expressed Tie2 controls angiogenesis and vessel maturation
Source: Nat Commun. 2017 Jul 18;8:16106. doi: 10.1038/ncomms16106 (PMC5520106; doi:10.1038/ncomms16106)
Supplement: Supplementary Information [file ncomms16106-s1.pdf]

File Name: Supplementary Information
Description: Supplementary Figures.

File Name: Peer Review File
Description:

Supplementary Figure 1

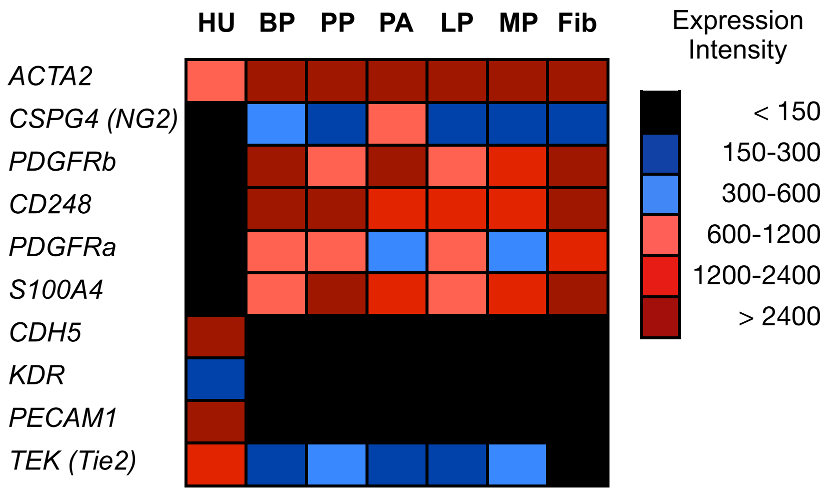

Supplementary Figure 1. Microarray-based screening of human brain pericytes (BP), placenta pericytes (PP), pancreas pericytes (PA), lung pericytes (LP), muscle pericytes (MP), human umbilical vein endothelial cells (HU) and human dermal fibroblasts (Fib) for pericyte, fibroblast and EC marker genes.

Supplementary Figure 2

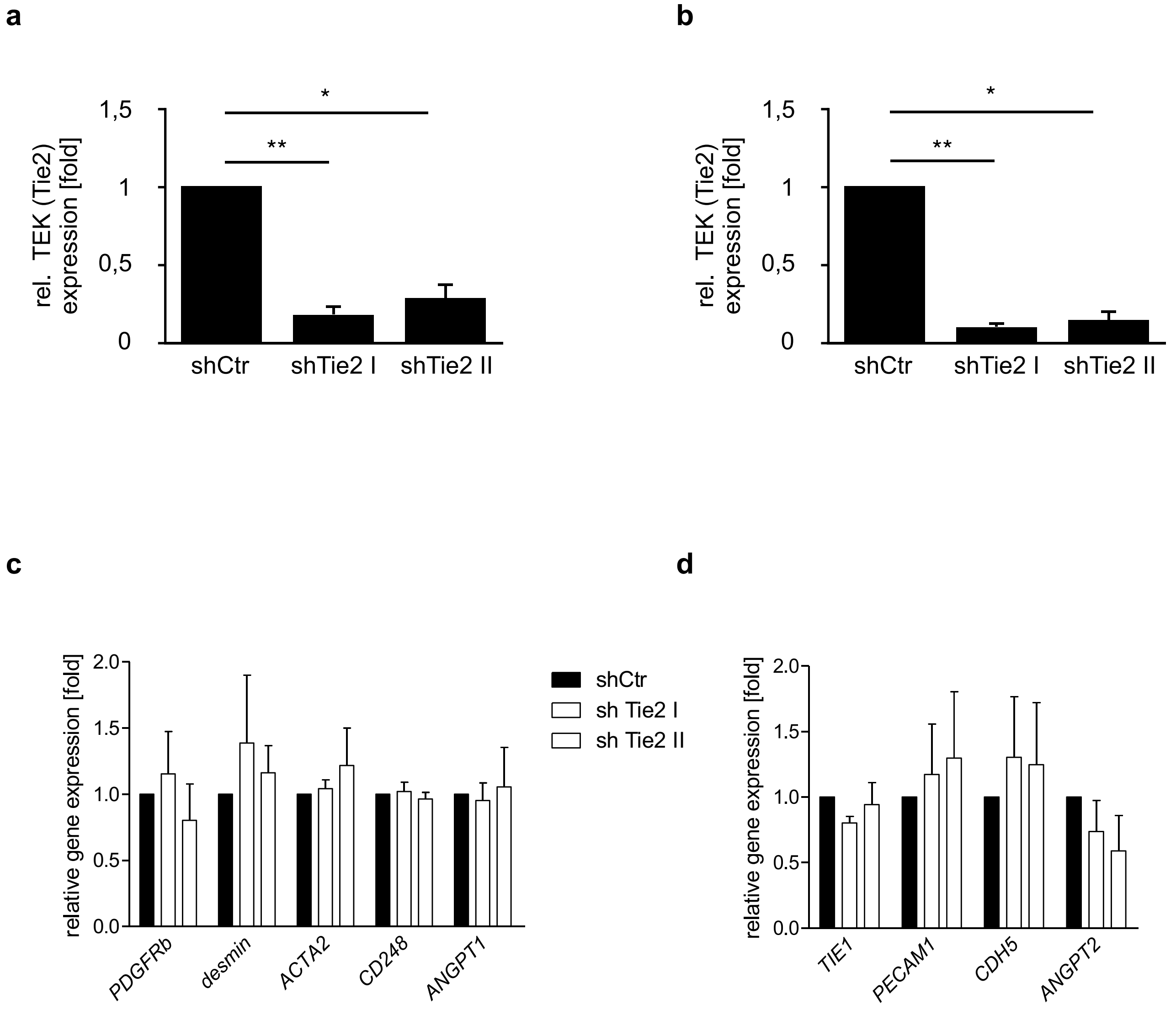

Supplementary Figure 2. (**a**) qPCR analysis of *TEK (Tie2)* expression in control (shCtr) and Tie2-silenced (shTie2 I/shTie2 II) BP (n=3). (**b**) qPCR analysis of *TEK (Tie2)* expression in control (shCtr) and Tie2-silenced (shTie2 I/shTie2 II) HUVEC (n=3). (**c**) qPCR analysis of common pericyte genes in control (shCtr) and Tie2-silenced (shTie2 I/shTie2 II) BP (n=5). (**d**) qPCR analysis of common endothelial genes in control (shCtr) and Tie2-silenced (shTie2 I/shTie2 II) HUVEC (n=3). Data are shown as mean±sd. Statistics were performed using One Way ANOVA. *P<0.05, **P<0.01.

Supplementary Figure 3

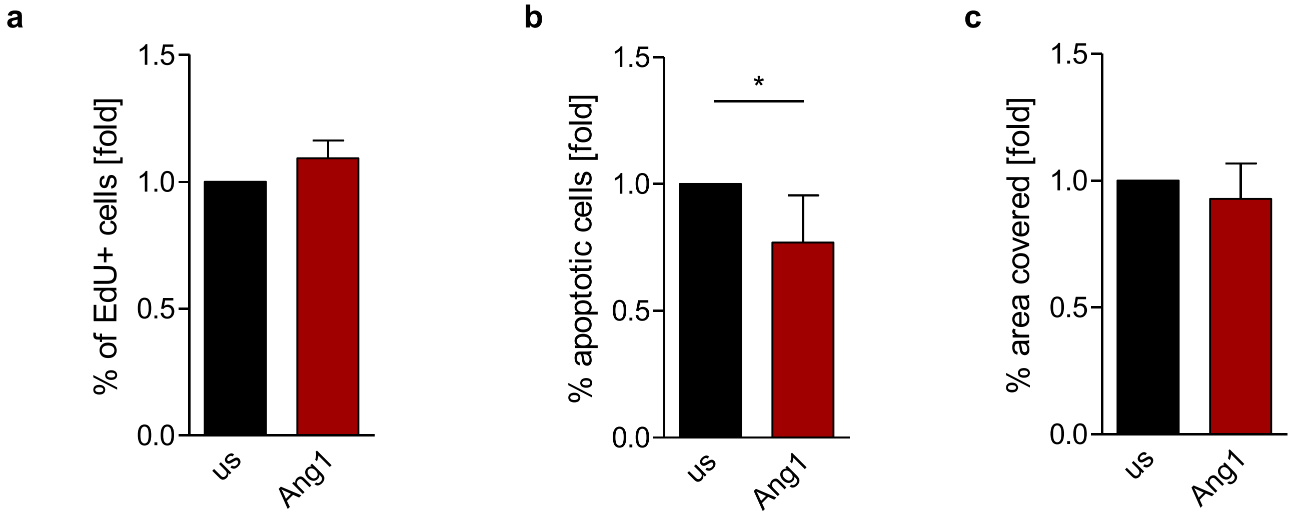

Supplementary Figure 3. Flow cytometric analysis of EdU incorporation (**a**), Annexin V staining (**b**) and lateral migration analysis in scratch assay (**c**) of human brain pericytes stimulated for 24h with recombinant human Ang1 (n=3-7). Data are shown as mean±sd. Statistics were performed using Student's t-test. *P<0.05.

Supplementary Figure 4

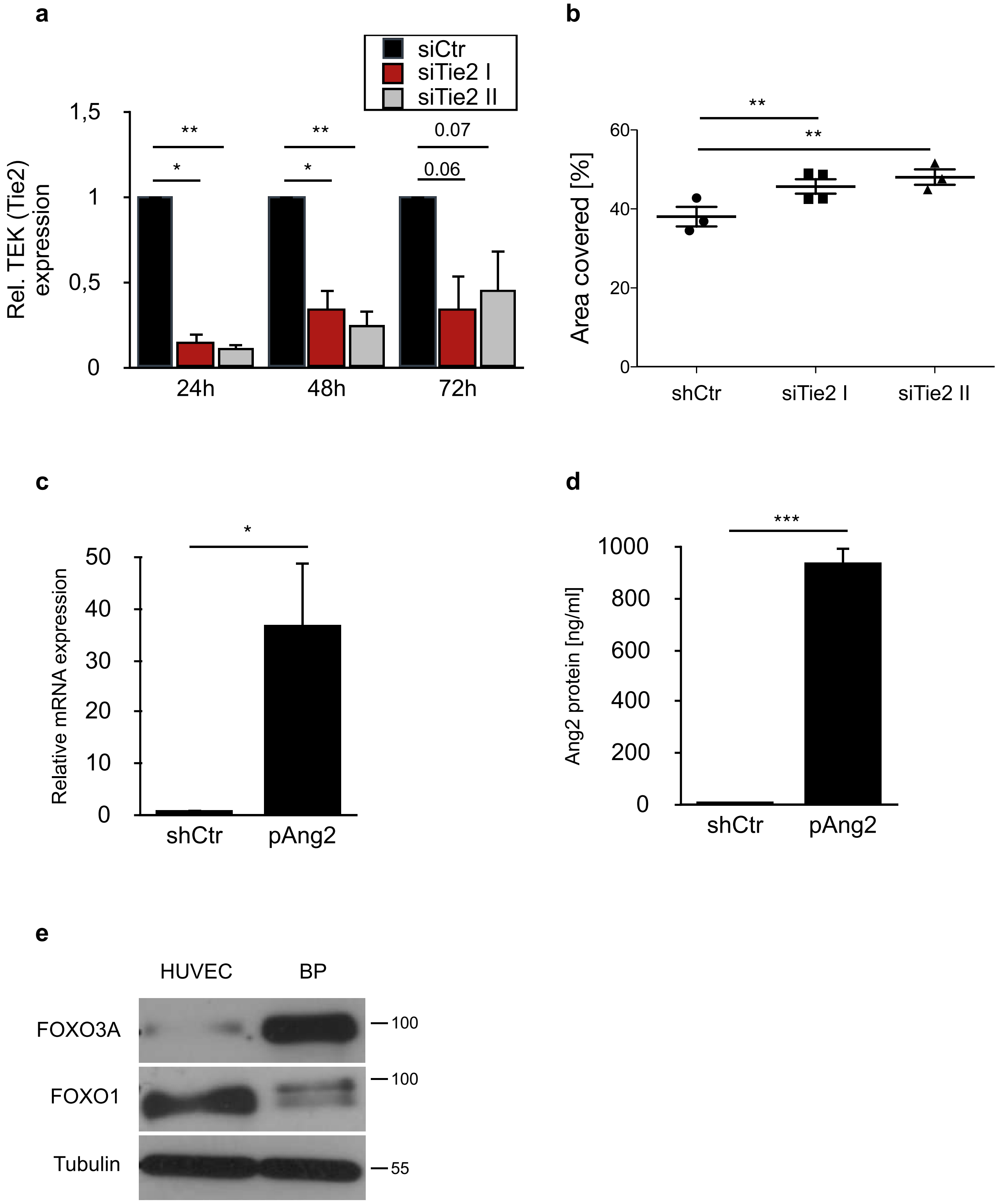

Supplementary Figure 4. (**a**) qPCR analysis of *TEK (Tie2)* expression in control (siCtr) and Tie2-silenced (siTie2 I/siTie2 II) BP (n=3). (**b**) Quantification of covered area by BP depleted of Tie2 (siTie2 I/ siTie2 II) or not depleted (siCtr) in a lateral scratch wound assay after 6.5h normalized to 0h. (**c**) qPCR analysis of *ANGPT2* expression in control (Ctr) and Ang2-overexpressing (pAng2) HUVEC (n=3). (**d**) ELISA analysis of Ang2 in control (Ctr) and Ang2-overexpressing (pAng2) HUVEC (n=2). (**e**) Western blot analysis of FOXO3A and FOXO1 expression in HUVEC and BP. Tubulin served as a loading control. Representative Western blot images are cropped versions and original images can be found in Supplementary Fig. 16. Data are shown as mean±sd. Statistics were performed using One Way ANOVA (a,b) and Student's t-test (c,d). *P<0.01, **P<0.01, ***P<0.001.

Supplementary Figure 5

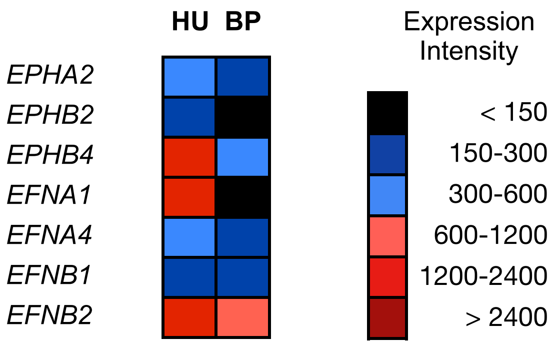

Supplementary Figure 5. Microarray-based expression screening of human brain pericytes (BP) and human umbilical vein endothelial cells (HU) for *EPH* receptors and ephrin ligands (*EFN*).

Supplementary Figure 6

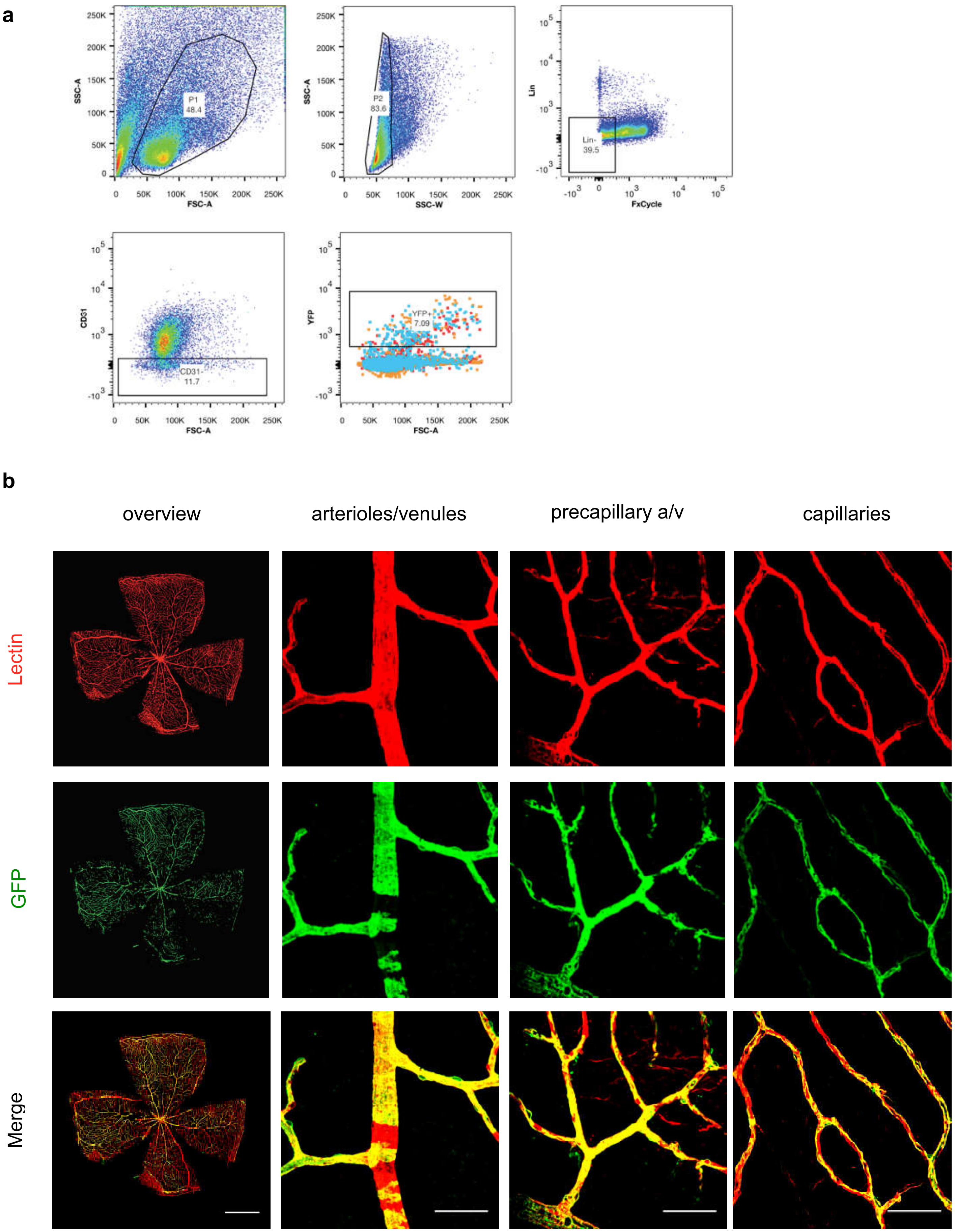

Supplementary Figure 6. (**a**) Schematic visualization of the strategy used for the isolation of Ng2-positive cells by FACS. (**b**) Representative images of the total retinal vasculature (postnatal day 6, P6), arterioles/venules, precapillary arteries/postcapillary venules or capillaries stained for GFP and with isoectin B4 in *Ng2-mT/mG* mice. Scale bars: 30 µm (b).

Supplementary Figure 7

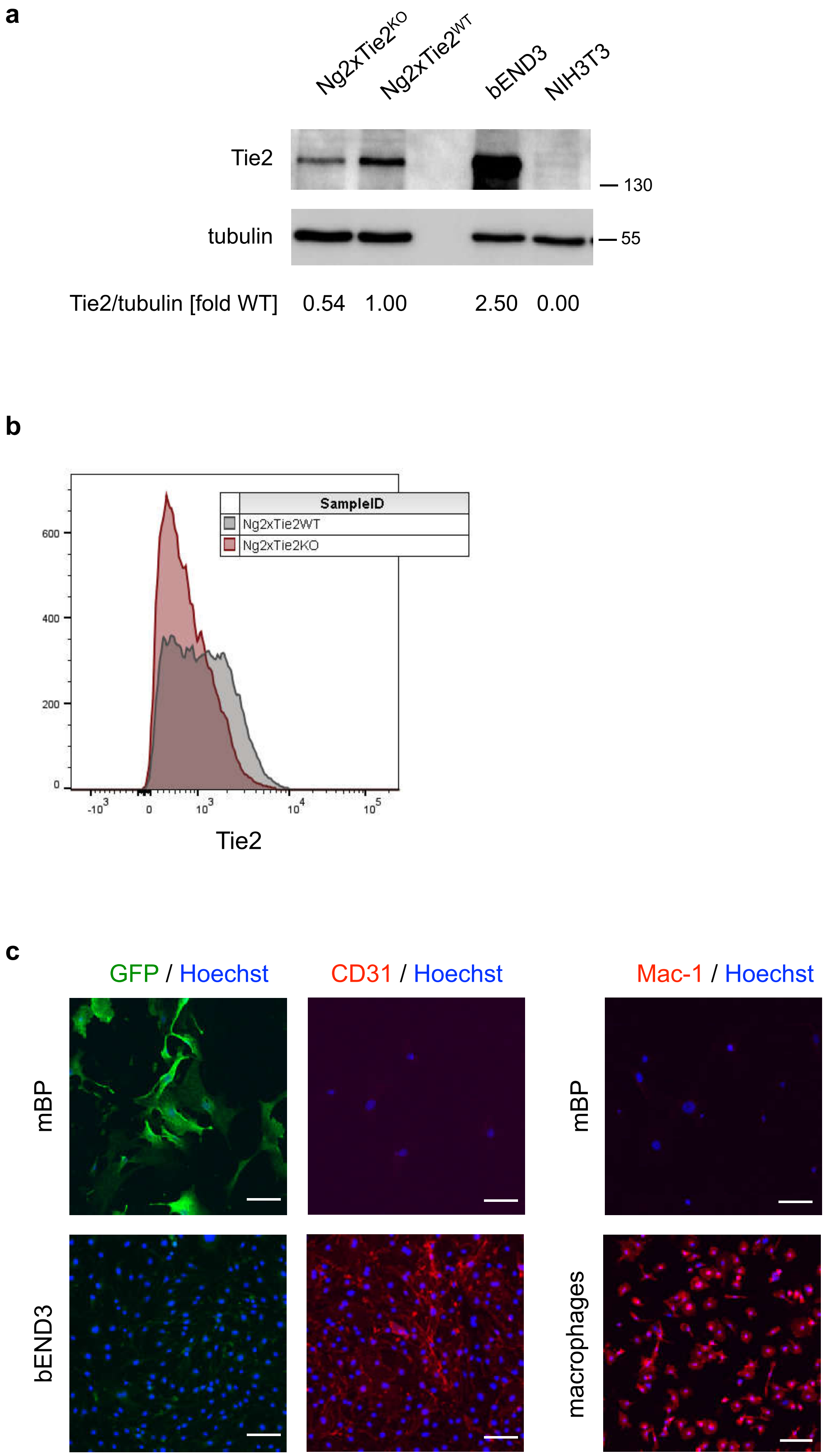

Supplementary Figure 7. Western blot (**a**), flow cytometric analysis of Tie2 (**b**) and representative stainings (**c**) of mouse brain pericytes (mBP) isolated from WT and *Tie2^{PEKO}* mice. Mouse brain endothelial cells (bEND3), fibroblasts (NIH3T3) and peritoneal macrophages were used as controls. Representative Western blot images are cropped versions and original images can be found in Supplementary Fig. 16. Scale bar: 100 µm (c).

Supplementary Figure 8

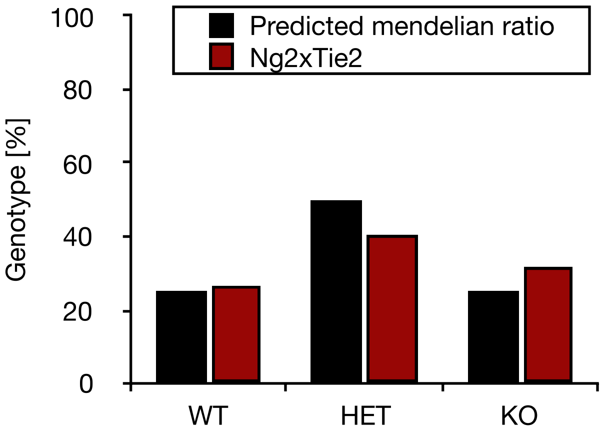

Supplementary Fig. 8. Genotype analysis of WT mice, heterozygotes (HET) or null mice (KO) for the *Ng2-Tie2* allele compared to predicted Mendelian ratio. Data are shown as mean.

Supplementary Figure 9

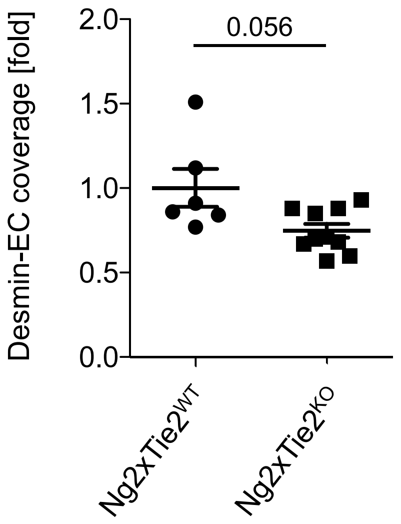

Supplementary Figure 9. Quantitative analysis of EC-pericyte coverage between CD31-positive vessels and desmin-positive pericytes in B16 tumours grown in *Tie2*^{PEKO} mice normalized to WT average. Data are shown as mean±sd. Statistics were performed using Mann-Whitney U test.

Supplementary Figure 10

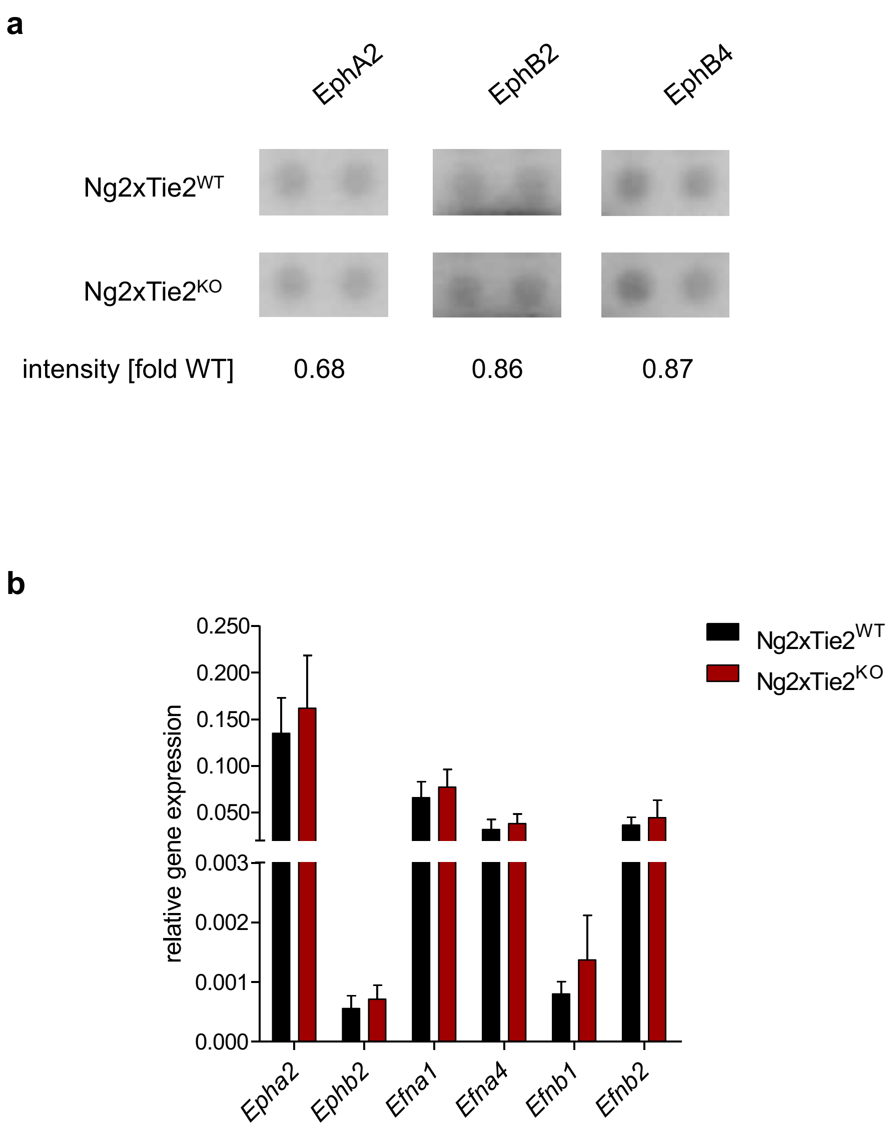

Supplementary Figure 10. (**a**) Mouse phospho-receptor tyrosine kinase array of whole tumour protein lysates from *Ng2-Tie2* animals. (**b**) qPCR analysis of whole tumor RNA lysates from *Ng2-Tie2* mice (n=7). Representative array images are cropped versions and original images can be found in Supplementary Fig. 18.

Supplementary Figure 11

a

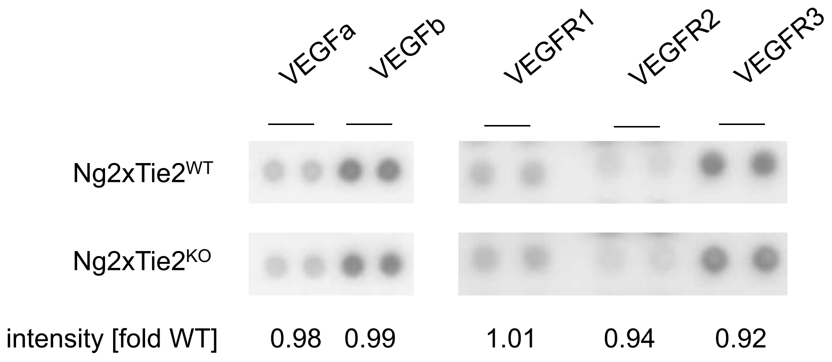

b

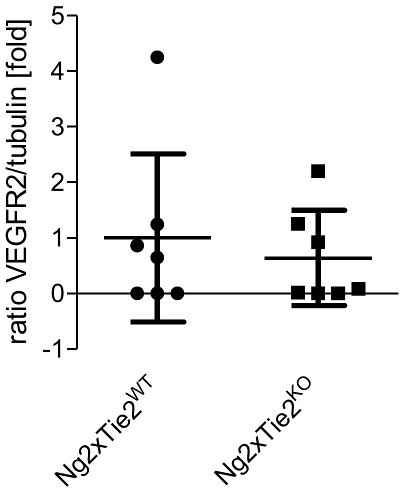

c

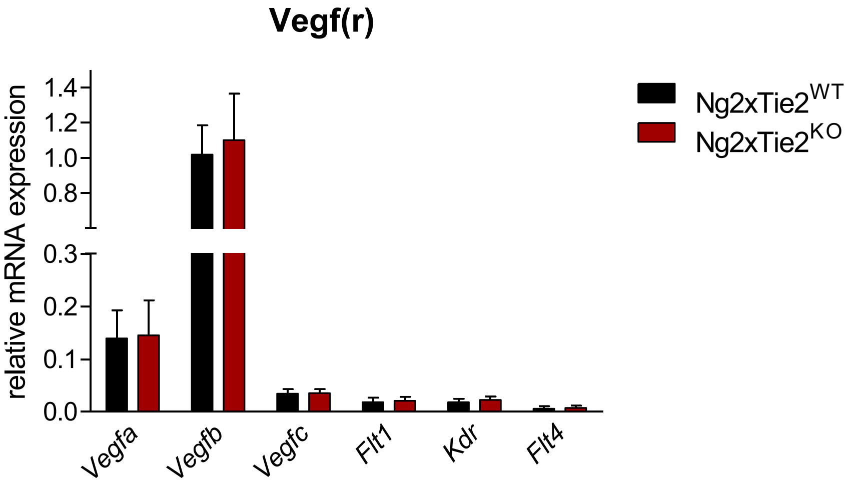

Supplementary Figure 11. (**a**) Mouse angiogenesis (left panel) and phospho-receptor tyrosine kinase profiler (right panel) of whole tumour protein lysates of *Ng2-Tie2* mice. (**b**) Ratio of VEGFR2 receptor protein levels of whole tumour protein lysates of *Ng2-Tie2* mice. (**c**) qPCR analysis of Vegf ligands and receptors in whole tumor RNA lysates and tumour protein lysates of *Ng2-Tie2* mice (n=7). Representative array images are cropped versions and original images can be found in Supplementary Fig. 18 and 19. Data shown as mean±sd.

Supplementary Figure 12

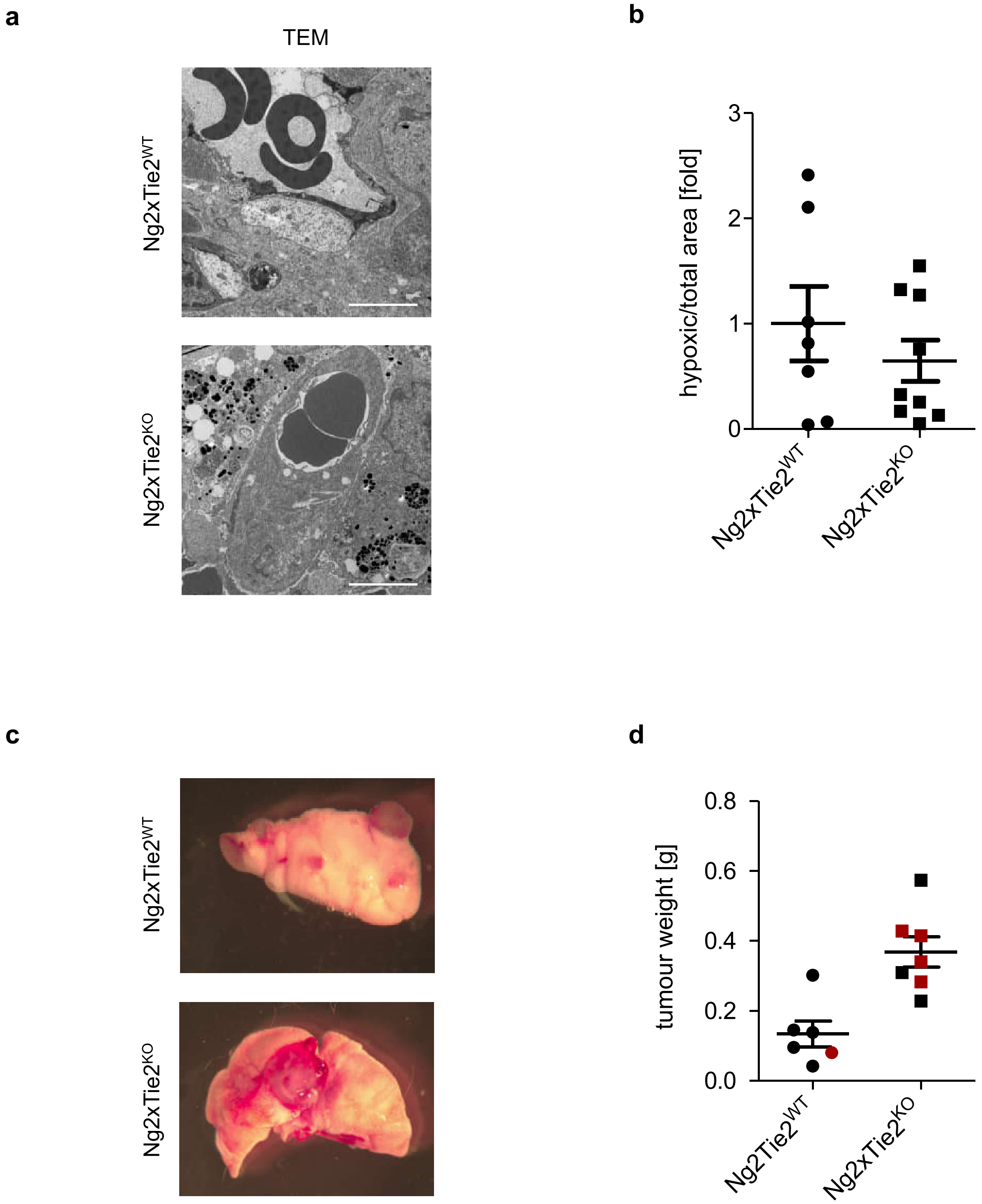

Supplementary Figure 12. (**a**) Transmission electron microscopy (TEM) of tumour vessels in WT and $Tie2^{PEKO}$ animals. (**b**) Quantification of the hypoxic area using Hypoxy Probe Kit. (**c**) Representative images of lungs of WT and $Tie2^{PEKO}$ animals upon surgical removal of the primary tumour. (**d**) Tumour weight in WT and $Tie2^{PEKO}$ mice. Red coloured dots/squares indicate animals showing metastasis in the lung. Scale bar = 5 µm (a). Data are shown as mean±sd.

Supplementary Figure 13

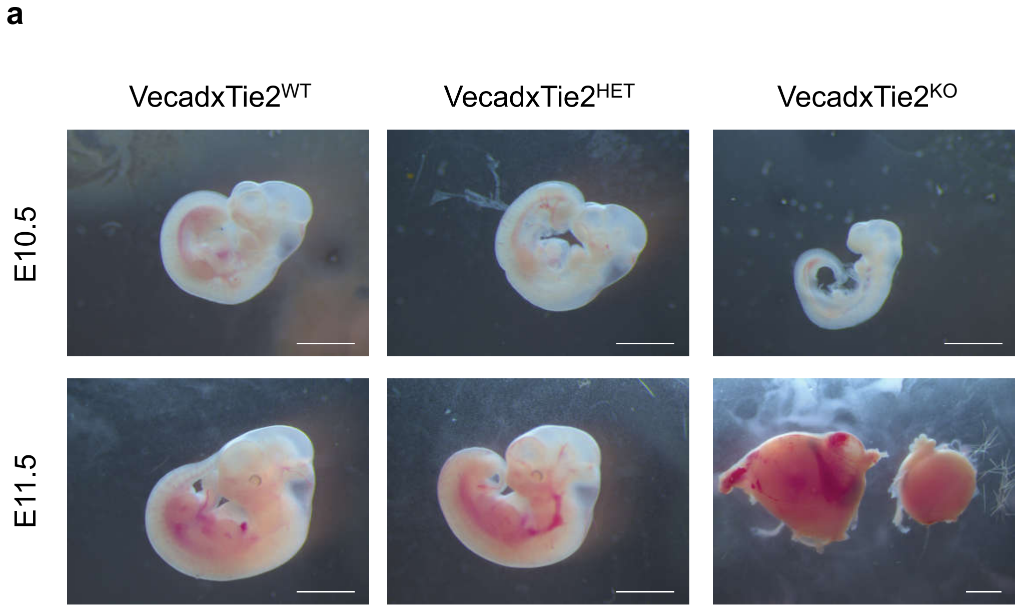

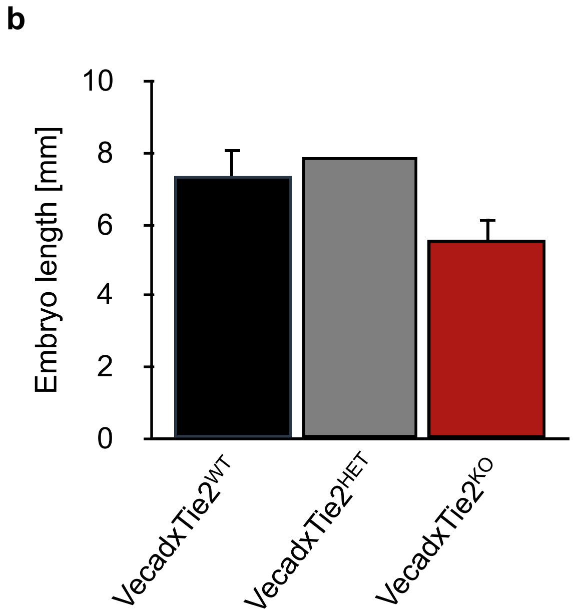

Supplementary Figure 13. Representative images of one litter of *Vecad-Cre-Tie2* mice at E10.5 and E11.5 **(a)** and quantification of embryo length at E10.5 (**b**). At E11.5 *Vecad-Tie2*^{KO} can not be isolated and just remaining placental resorptions were found. Correspondingly, genotyping revealed no birth of knockout animals. Scale bars: 2 mm (a). Data are shown as mean±sd.

Supplementary Figure 14

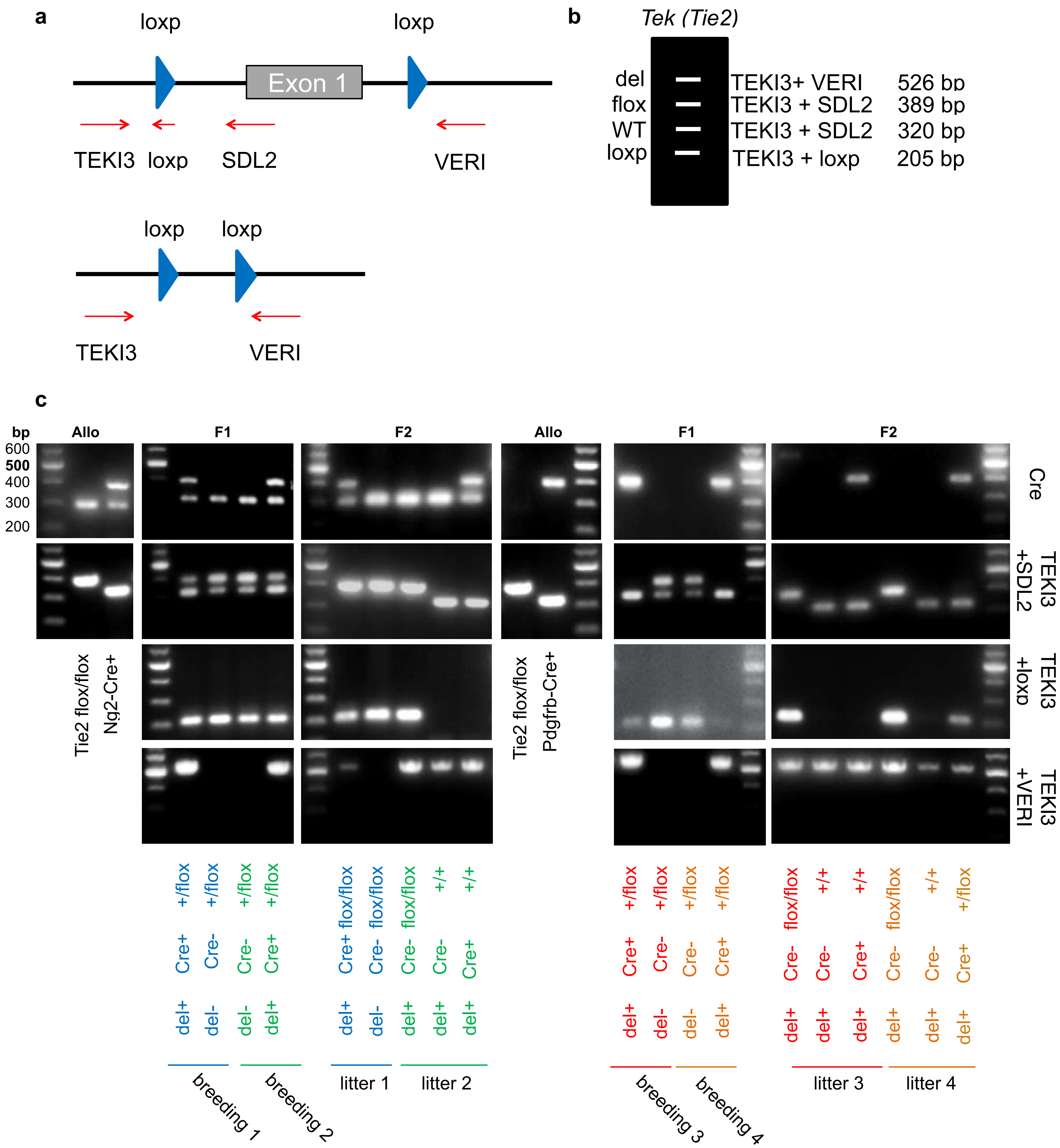

Supplementary Figure 14. (**a**) Schematic targeting strategy of the Tek (*Tie2)* locus and the corresponding binding sites of the three used genotyping primers (TEKI3, SDL2, VERI). (**b)** Schematic overview of the genotyping bands. (**c**) Genotyping of the different generations (Allomating, F1, F2) during the breeding of *Ng2-Tieflox/flox* (left panel) and *Pdgfrb-Tie2flox/flox* mice. Crossing with *Ng2-Cre* shows activity in the male germ line (breeding 2 and litter 2) seen by Cre-negative and Tie2 WT mice showing a deletion (del) band. However, *Ng2-Cre* is not active in the female germline (breeding 1 and litter 1). *Pdgfrb-Cre* is active in the male and female germline as shown in breeding 1 and 2 of Cre-positive males or females resulting in litter 1 and 2. In both scenarios Cre-negative and WT offspring show a band of Tie2 deletion.

Supplementary Figure 15

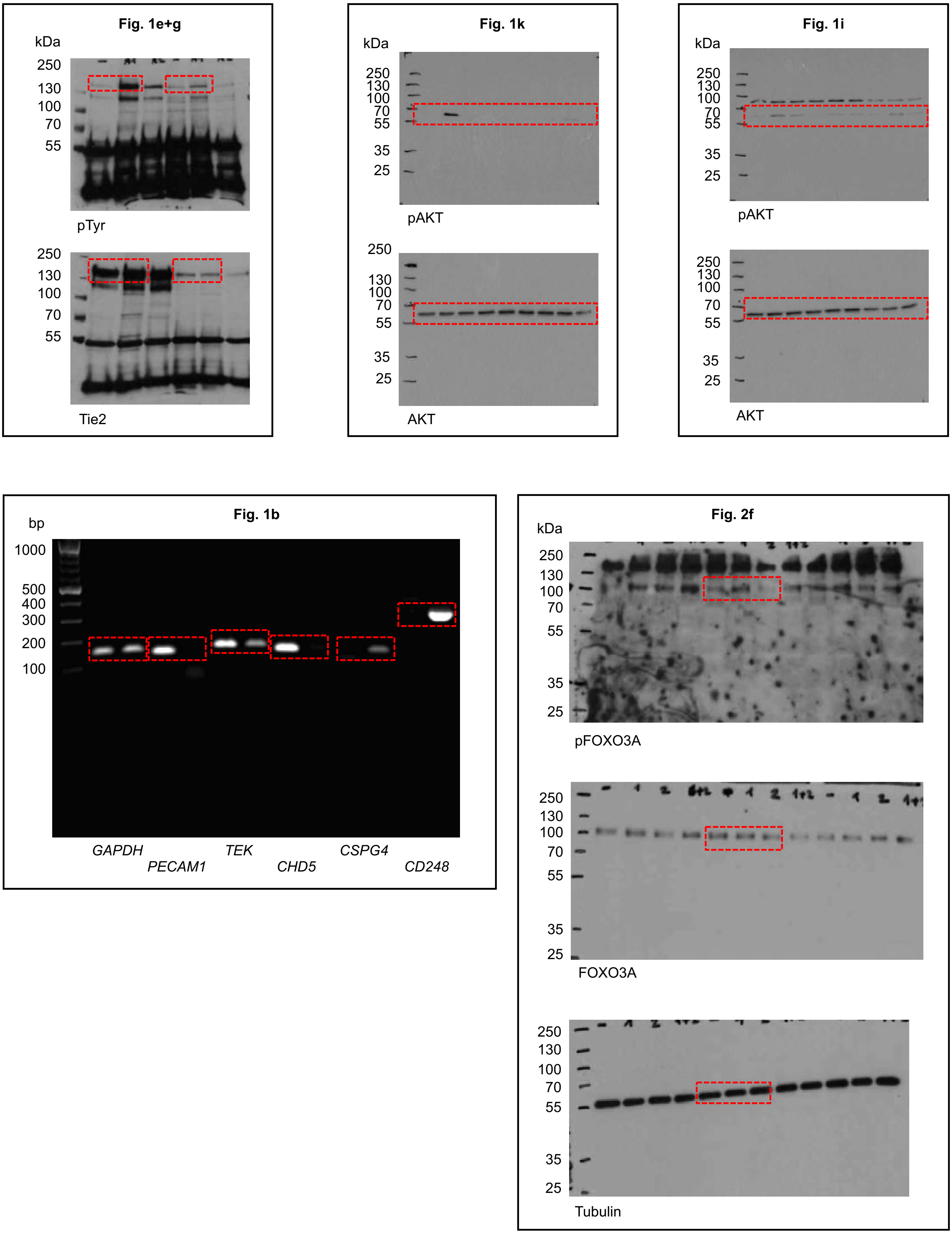

Supplementary Figure 15. Uncropped blots of Fig. 1e, g, i, and k, Fig 2f. Uncropped gel of Fig. 1b.

Supplementary Figure 16

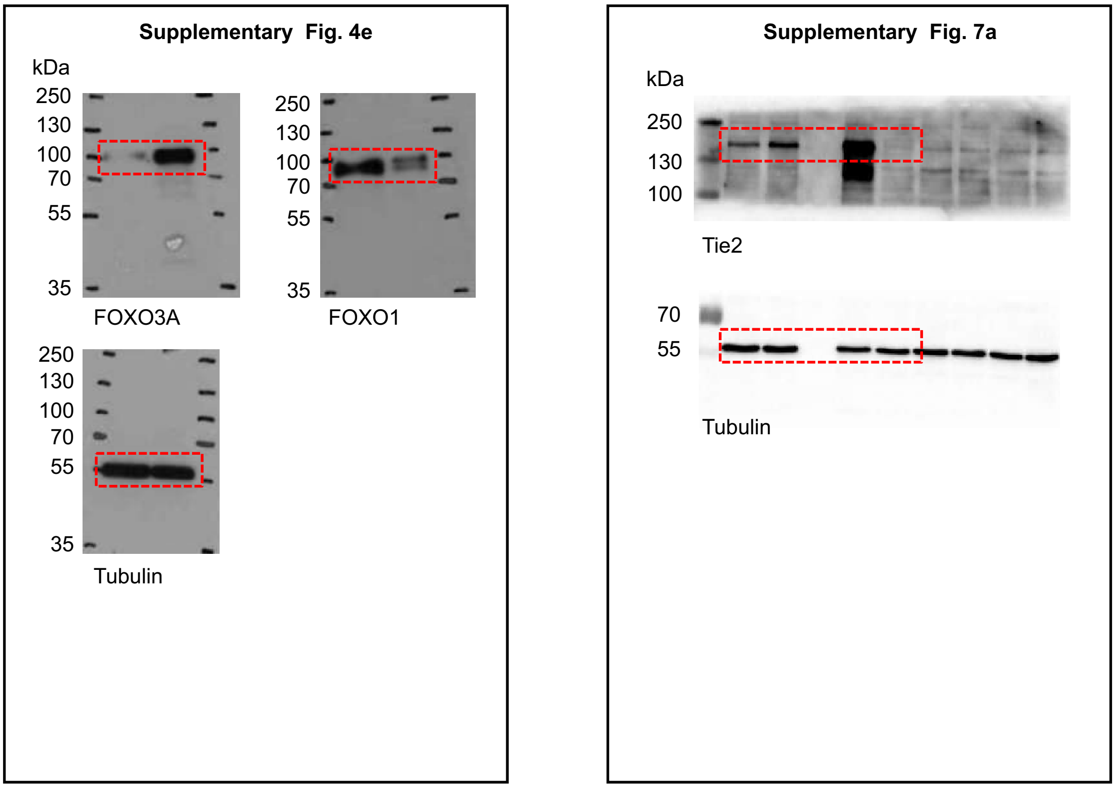

Supplementary Figure 16. Uncropped blots of Supplementary Fig. 4e and 7a.

Supplementary Figure 17

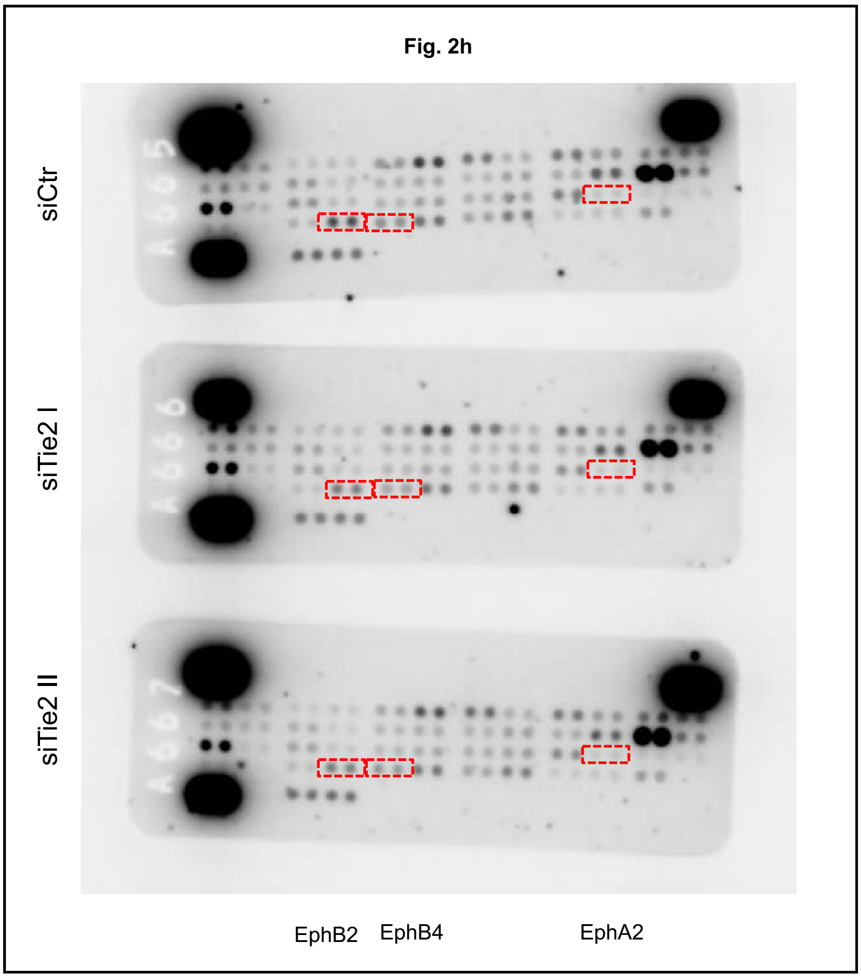

Supplementary Figure 17. Uncropped human phospho-receptor tyrosine kinase array of Fig. 2h.

Supplementary Figure 18

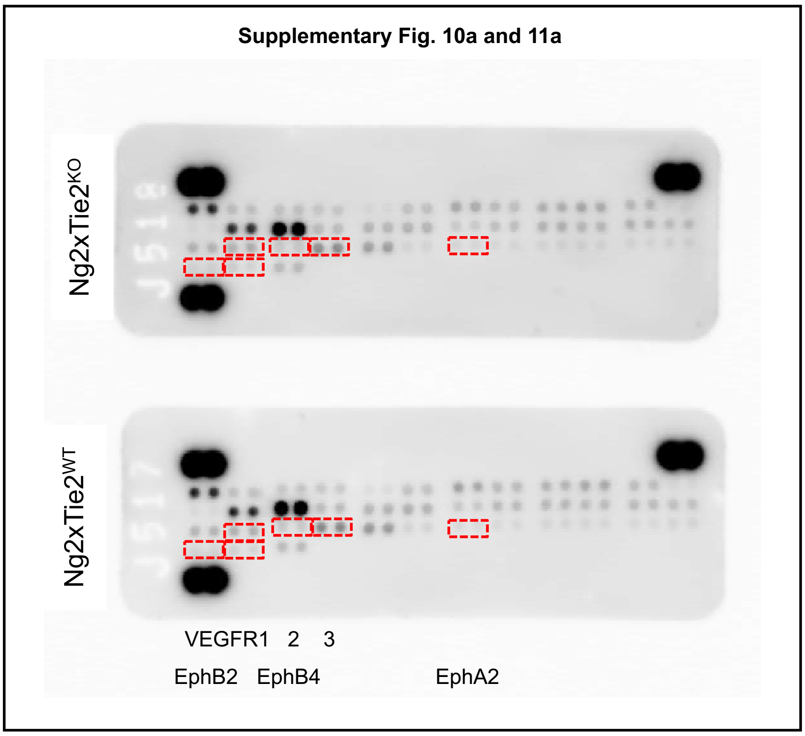

Supplementary Figure 18. Uncropped mouse phospho-receptor tyrosine kinase array of Supplementary Fig. 10a and 11a.

Supplementary Figure 19

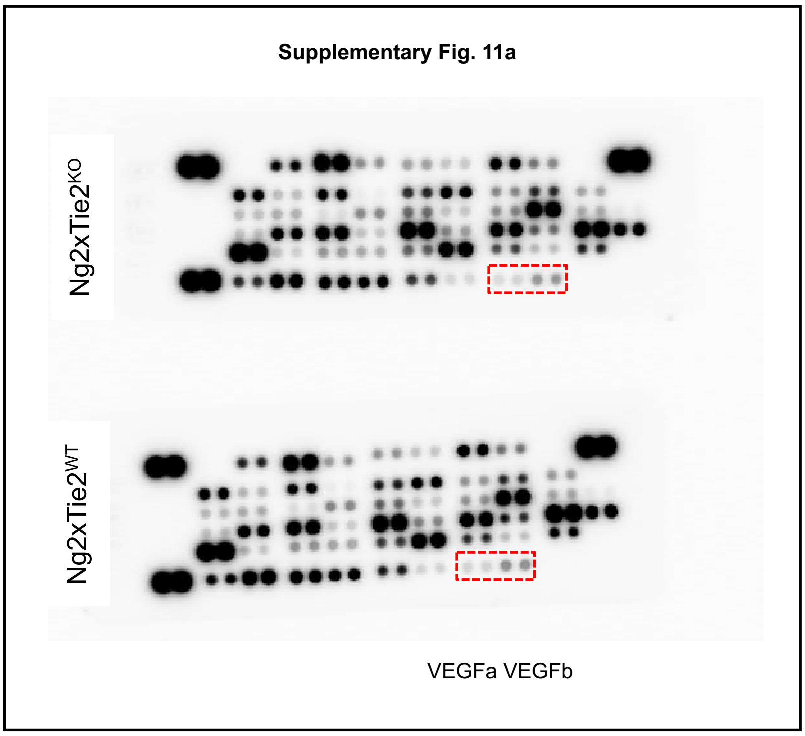

Supplementary Figure 19. Uncropped mouse angiogenesis array of Supplementary Fig. 11a.
